# Microwaves in clean energy technologies

**DOI:** 10.1098/rsta.2024.0394

**Published:** 2025-05-22

**Authors:** Samuel Hefford, Michael Barter, M. Usman Azam, Bhupinder Singh, Georgios Dimitrakis, Xiangyu Jie, Peter Edwards, Daniel R. Slocombe

**Affiliations:** ^1^ Cardiff University, Cardiff, UK; ^2^ University of Nottingham, Nottingham, UK; ^3^ Queen Mary University of London, London, UK; ^4^ University of Oxford, Oxford, UK

**Keywords:** microwaves, hydrogen, green energy, batteries, plastics, ammonia

## Abstract

Energy in the microwave spectrum is increasingly applied in clean energy technologies. This review discusses recent innovations using microwave fields in hydrogen production and synthesis of new battery materials, highlighting the unique properties of microwave heating. Key innovations include microwave-assisted hydrogen generation from water, hydrocarbons and ammonia and the synthesis of high-performance anode and cathode materials. Microwave-assisted catalytic water splitting using Gd-doped ceria achieves efficient hydrogen production below 250°C. For hydrocarbons, advanced microwave-active catalysts Fe–Ni alloys and ruthenium nanoparticles enable high conversion rates and hydrogen yields. In ammonia synthesis, microwaves reduce the energy demands of the Haber–Bosch process and enhance hydrogen production efficiency using catalysts such as ruthenium and Co_2_Mo_3_N. In battery technology, microwave-assisted synthesis of cathode materials like LiFePO_4_ and LiNi_0.5_Mn_1.5_O_4_ yields high-purity materials with superior electrochemical performance. Developing nanostructured and composite materials, including graphene-based anodes, significantly improves battery capacities and cycling stability. The ability of microwave technology to provide rapid, selective heating and enhance reaction rates offers significant advancements in clean energy technologies. Ongoing research continues to bridge theoretical understanding and practical applications, driving further innovations in this field. This review aims to highlight recent advances in clean energy technologies based upon the novel use of microwave energy. The potential impact of these emerging applications is now being fully understood in areas that are critical to achieving net zero and can contribute to the decarbonization of key sectors. Notable in this landscape are the sectors of hydrogen fuel and battery technologies. This review examines the role of microwaves in these areas.

This article is part of the discussion meeting issue ‘Microwave science in sustainability’.

## 1. Introduction

Energy in the microwave spectrum is used in many disciplines, and recent developments in our understanding of microwave interactions with feedstock and catalyst materials have led to many emerging applications in clean energy technologies. In this review, we provide a comprehensive discussion focusing upon microwaves for hydrogen energy and new generation battery technologies. The review targets the steadily increasing market of sustainable energy sources. Coupled with the importance of sustainable circular economies, microwave-assisted reactions are now showing great potential in materials synthesis and process intensification.

Microwaves provide unique advantages for activation and interrogation of chemical processes. The potential to fundamentally control the mechanism of heating using microwave electric and microwave magnetic fields independently in addition to an enhanced understanding of microwave heating in complex mixtures has led to new opportunities in sustainable energy. Here, we survey innovations in hydrogen generation from water, hydrocarbons and ammonia, and look at the manufacture of high-performance anode and cathode materials.

Energy in the microwave spectrum has played a pivotal role in many technologies since first being generated in the lab in the nineteenth century. Their versatile applications span across measurement and heating to stimulating phenomena such as magnetic and cyclotron resonance, catalysis, sintering and synthesis. Low-power microwave electric fields have been instrumental in sensitively measuring changes in charge dynamics from diverse phenomena like catalyst deactivation and ammonia adsorption in zeolites [1,2]. High-frequency magnetic fields find common usage in advanced imaging and resonance techniques, including magnetic resonance imaging, nuclear magnetic resonance and electron paramagnetic resonance [3,4]. More recently, microwave magnetic fields have produced groundbreaking results, such as quantum imaging of current flow in graphene and neuronal pulse measurement [5,6].

The utilization of microwaves at higher power levels has been shown to significantly influence organic reactions, synthesis and catalysis [7–9]. Unlike conventional heating, microwave heating mechanisms produce distinctly different outcomes due to the rapid and selective generation of heat in regions characterized by varying complex permittivity. This interaction activates chemical processes and can lead to complex phenomena such as field ionization, which can subsequently alter reaction pathways.

Microwave heating has shown significant promise in clean energy technologies, yielding enhanced reaction rates, lower temperatures and higher yields. However, understanding these benefits remains a challenge, as theoretical foundations often lag behind experimental results. This gap has spurred debate and progress in understanding microwave interactions with complex chemical systems. Over the past two decades, there have been substantial advancements in understanding the interactions between microwaves and chemical species. These advancements have facilitated the development of theoretical frameworks that elucidate microwave-enhanced chemical reactions [10,11]. Major steps have also been made in the assessment of temperature in microwave reactions, with in-depth studies revealing the differences between measured temperature values and the environment at reaction sites [12]. Here, we review representative results and progress based upon a rapidly advancing knowledge in this field, which has led to growing innovation in clean energy technologies.

In this review, we discuss applications of microwave heating as well as processing of materials using microwave plasmas. For discussion on the fundamentals of these processes, we would like to point the reader to the following resources [13] and [14] for further explanation of microwave heating processes and microwave plasmas, respectively.

## 2. Microwaves in new hydrogen energy technologies

### (a) Hydrogen from water

In recent work, the advantageous effects of microwave heating were demonstrated in low-temperature, catalytic water splitting to produce hydrogen using microwaves [15], highlighting the role of reduced oxides in the process. Most global hydrogen production comes from methane reforming, which emits large amounts of CO_2_. Producing hydrogen from water using green energy is a sustainable alternative, but it is energy-intensive and requires higher temperatures.

Researchers at Universitat Politècnica de València reported a microwave-assisted method using oxide catalysts to release hydrogen from water at low temperatures [15]. Microwave irradiation causes highly localized heating, leading to very different results compared with conventional heating methods. The researchers used Gd-doped ceria (CGO) in a two-step process: microwave treatment partially reduces CGO, creating oxygen vacancies and increasing conductivity; the material is then re-oxidized with water to form H_2_. The process occurs below 250°C, though local temperatures may be higher. This localized heating intensifies the process while maintaining a lower bulk temperature. An increase in catalyst conductivity, induced by microwave heating, plays a key role in hydrogen release. The concept of an induction temperature (approx. 110°C) where energy absorption increases, leading to thermal runaway, is proposed by the authors. Oxygen vacancies increase conductivity and as a consequence, heating rates in microwaves are increased, enhancing material activation.

Thermodynamic simulations revealed that the exothermic enthalpy difference between oxygen-vacancy generation and water dissociation supports the endothermic reduction cycle. Dopant type and strength influence the reaction’s energy requirements. Serra, Catalá-Civera and their team also compared operating energy costs with conventional H_2_ production methods, suggesting microwave technology could become competitive with more established technologies such as electrolysis. Selective use of microwave energy to reduce oxides is key to optimizing efficiency. This innovation has led to an important area of research in hydrogen generation, but further studies are needed to improve the selective microwave reduction.

One month after the groundbreaking publication of water splitting using microwaves and CGO catalysts, another group published results showing hydrogen generation by two-step thermochemical cycles [16]. The study investigated a new method for continuous hydrogen regeneration using two-step water splitting, with both thermal reduction and water-splitting steps performed under the same microwave irradiation power. The high entropy oxide system (HEOS) achieved a maximum hydrogen generation rate of 13.89 ml min^−1^ g^−1^ at 700 W microwave power, with a maximum hydrogen yield of 122 ml g^−1^. The authors claim that these results surpass the thermodynamic limits of current materials like spinel ferrites and ceria.

The authors suggest the presence of microwave plasma discharges in the HEOS, enhancing the water-splitting process, but also accelerating material sintering. Despite this, the discharge process is uniform and reproducible, providing a basis for optimizing discharge intensity and pulse properties through morphological design. The short O_2_ generation times (4 min) under microwave irradiation and the efficient energy transfer compared with conventional thermal reduction and water-splitting processes are central to the improved efficiencies. While most reactor designs for such reactions have used solar concentrators, the growing availability of low-cost carbon-free electricity makes localized microwave heating a viable option for thermochemical water splitting.

More recently, work has followed in which authors evaluate techno-economic aspects of water splitting with microwaves, considering the economic feasibility of technically available designs [17]. The authors consider two thermochemical water-splitting systems, comparing methods using solar concentrators with microwave thermochemical water splitting at lower measured temperatures. Optimum geometries of printed circuit heat exchangers are proposed considering the cost analysis. The levelized cost of hydrogen (LCOH) and system efficiency were calculated for the solar concentrator and the system using microwaves. Although the use of microwaves showed lower efficiency than the conventional method with solar heating, it yielded more optimized LCOH. Additionally, an innovative use of heat recuperation systems is proposed, in which the authors suggest that they can avoid 10−20% of the cost for both systems.

### (b) Hydrogen from hydrocarbons

Hydrocarbons have been explored as a hydrogen carrier, including light fractions such as methane, through to diesel fractions, waxes and even polymers. It has been proposed that these hydrocarbons offer a promising material for hydrogen storage, providing a safe and efficient method for hydrogen release through microwave-assisted catalytic decomposition.

Microwave-assisted heating efficiently converts methane into syngas through catalytic and plasma pathways. Gangurde *et al*. compared conventional and microwave-assisted hydrothermal methods to synthesize ruthenium-doped strontium titanate (SrTiO_3_) perovskite catalysts [18]. They found that microwave irradiation of SrTiO_3_ catalysts efficiently converted CH_4_ and CO_2_ into syngas, achieving an H_2_/CO ratio of 0.9. Additionally, microwave plasma technology demonstrated excellent performance in methane reforming without the need for catalysts. Li *et al*. used Fe_2_O_3_ as a catalyst to pyrolyse biomass, producing a large surface area, porous, Fe-rich biomass-derived carbon [19]. This material was then employed in microwave-assisted methane reforming, achieving high methane conversion at 800°C. The CH_4_ and CO_2_ conversion rates remained stable over 160 min, producing a syngas content of 88.79% with an H_2_/CO ratio of 0.92. Compared with traditional heating methods, microwave-assisted heating enhances chemical reactions and positively affects catalyst preparation. Czylkowski *et al*. combined steam reforming of methane with a microwave plasma source, optimizing the absorbed microwave power, gas composition and flow rate [20]. This method achieved a hydrogen production energy yield of 42.9 g H_2_ per kWh and operated stably at high gas flow rates.

Wang *et al*. investigated wet reforming of liquid-phase methane by directly coupling liquid-phase microwave discharge plasma [21]. At a microwave input power of 900 W, they achieved a methane conversion rate of 94.3% and a hydrogen concentration of 74.0%. Optimizing the electrode structure and improving plasma system stability resulted in higher hydrogen yield and energy efficiency, with a peak hydrogen production efficiency of about 0.92 mmol kJ^−1^.

Gonzalez-Cortes *et al*. explored the microwave-assisted catalytic decomposition of paraffin wax (C_26_H_54_) using ruthenium nanoparticles supported on carbon, achieving rapid hydrogen release [22]. Microwave-assisted catalysis using ruthenium nanoparticles embedded in paraffin wax yielded approximately 7 wt% hydrogen. Thermodynamic analysis showed that deep dehydrogenation reactions become more favourable with higher reaction temperatures and increased carbon nuclearity. The hydrogen concentration reached 80−60 mol%, compared with 40 mol% without the metal catalyst.

The authors found that the local temperature of the catalytic sites was significantly higher than the surface temperature due to microwave-induced hotspots. This non-equilibrium localized superheating accelerated the hydrogen release process. Lower absorbed power extended the reaction time but did not affect the gas composition markedly.

Jie *et al*. demonstrated the rapid liberation of high-purity hydrogen by microwave-promoted catalytic dehydrogenation of liquid alkanes using Fe and Ni particles supported on silicon carbide (SiC) [23]. The study achieved an H_2_ production selectivity of approximately 98% from all evolved gases, with minimal CO and CO_2_ (less than 0.5 vol%). High-purity hydrogen was produced from various liquid alkanes (C_9_ to C_17_), with the highest selectivity observed using a Fe−Ni alloy catalyst. The catalyst system maintained efficiency over multiple cycles, although hydrogen production and selectivity decreased slightly due to carbon deposition on the catalyst sites. Comparative experiments showed that microwave irradiation significantly enhances hydrogen production compared with conventional thermal dehydrogenation methods. SiC-supported catalysts, particularly those involving Fe–Ni alloys, demonstrated superior performance with high hydrogen selectivity and low by-product formation.

**Figure 1.**
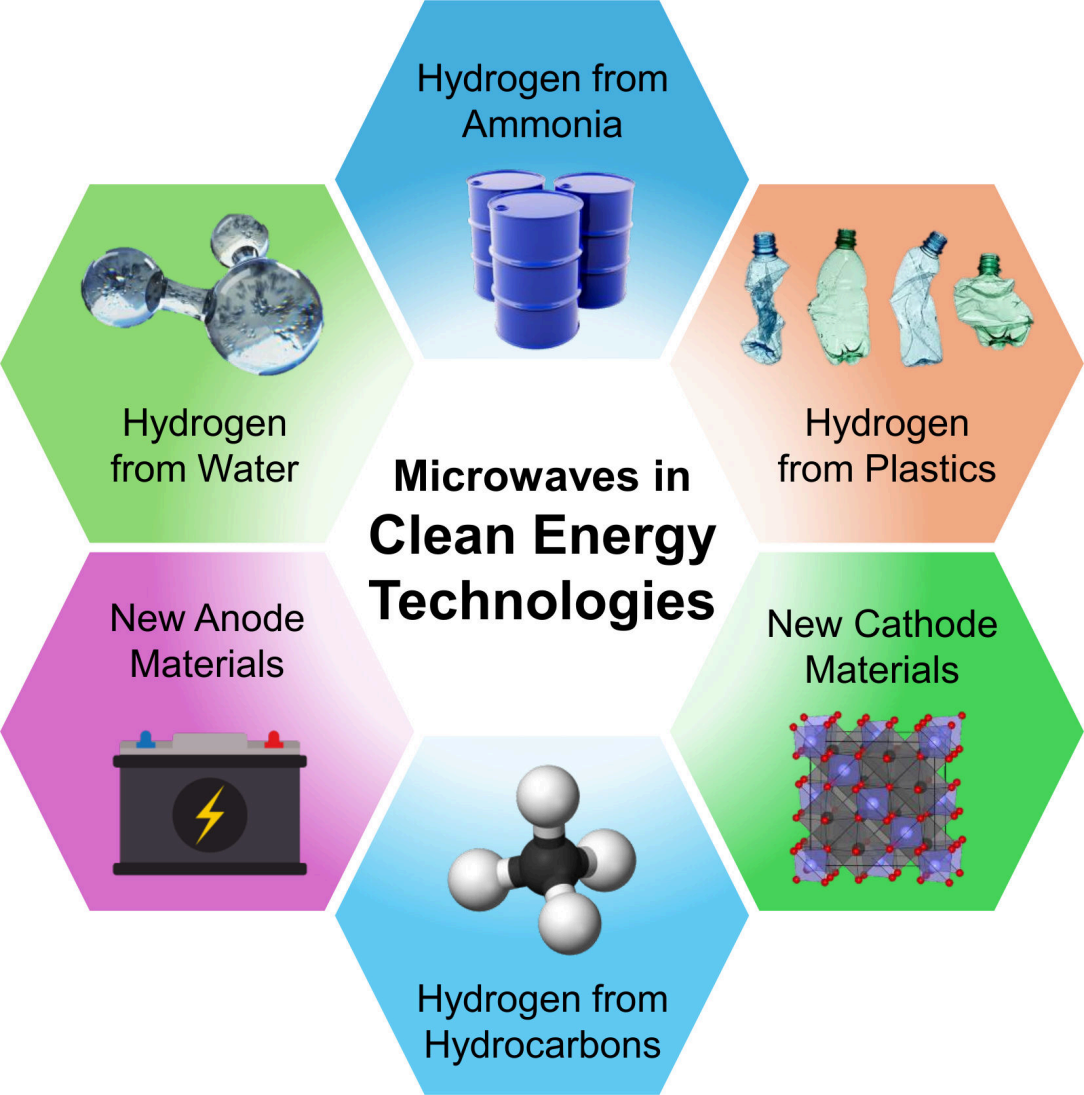
Microwaves in clean energy technologies.

**Figure 2.**
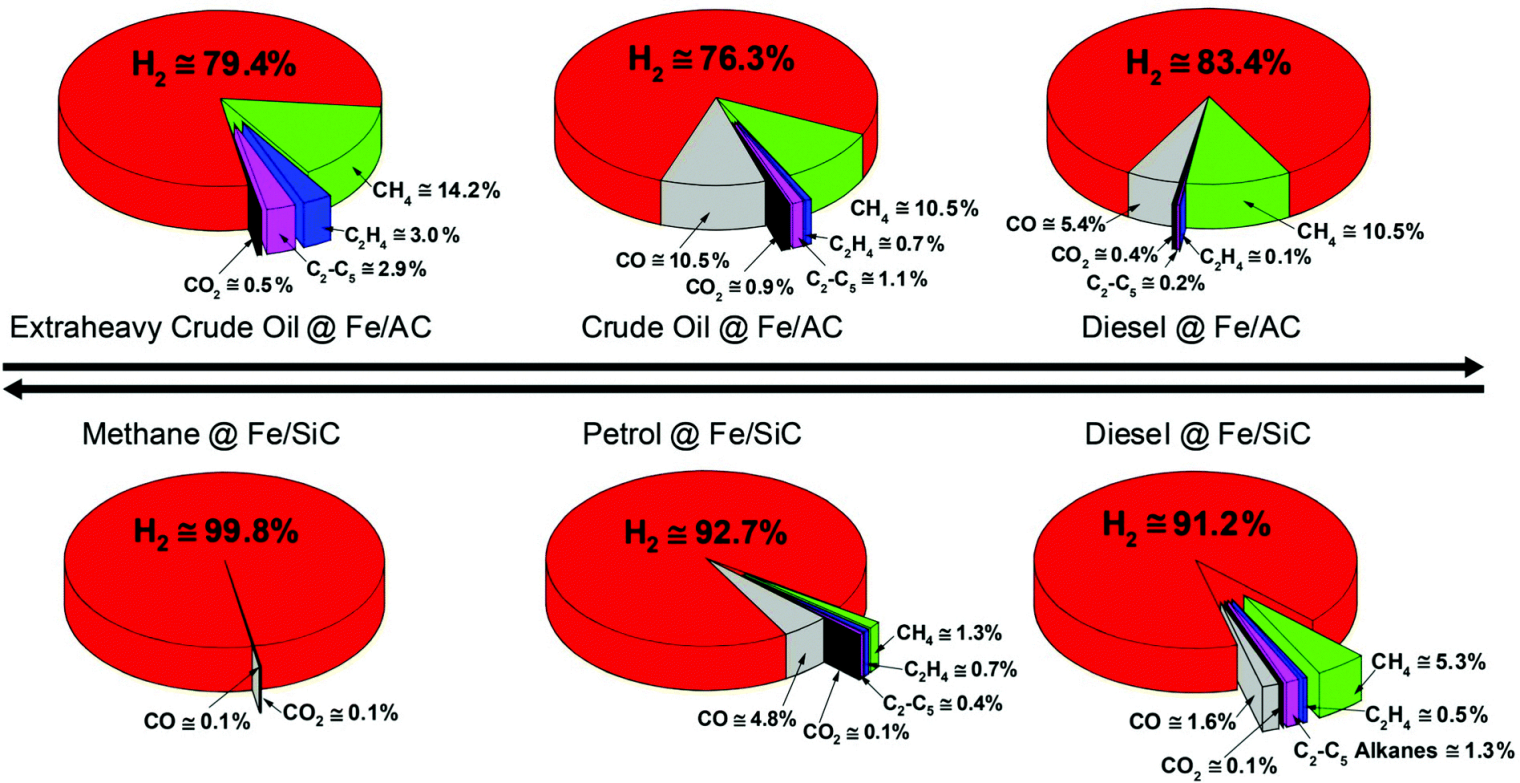
Microwave-initiated dehydrogenation of fossil hydrocarbon feedstocks [25].

In another study, Jie *et al*. investigated the interaction between microwave radiation and solid Fe catalysts, focusing on the dehydrogenation of hexadecane [24]. The findings reveal that the optimal particle size for Fe metal catalysts in microwave-initiated reactions is between 80 and 120 nm. This size range ensures that Fe particles are effective microwave absorbers, significantly enhancing catalytic performance. The effectiveness of these catalysts is strongly influenced by the ratio of the particle radius to the microwave skin depth (*r*/δ). The research also demonstrates that combining Fe metal particles with activated carbon (AC) results in synergistic effects, improving heating efficiency and overall catalytic activity. The study emphasizes that the physical size of metal catalyst particles decisively affects their heating and catalytic properties under microwave conditions. Additionally, Fe-based catalysts have been demonstrated to dehydrogenate a broad range of fossil hydrocarbons, from extra crude oil down to diesel, petrol and gaseous natural gas [25] (figures 1 and 2).

### (c) Hydrogen from plastics

Deconstruction of waste plastics into hydrogen is considered one of the most promising approaches, owing to its high energy density. However, the success of this process lies in the precise activation of C–H bonds, which requires high energy as compared with C–C bond cleavage [26]. Conventionally, it was done by the two-step process, starting with the thermal decomposition of plastics into hydrocarbons followed by catalytic steam reforming to produce hydrogen-rich gases [27]. However, the high-energy requirements and the significant generation of CO_2_ as a by-product limit the sustainability of the whole process, however, these issues could be addressed by the use of microwave heating. Careful consideration is required in developing catalysts with desirable catalytic and electromagnetic properties, as both are required to effectively promote the C–H bond scission and maximize the H_2_ yield.

Ding *et al*. [28] performed the microwave-assisted pyrolysis of low-density polyethylene (LDPE) over NiO-loaded HY at 500°C and reported a gas yield of approximately 50% with 35 vol% of H_2_. Despite this, the process resulted in a significant yield of higher hydrocarbons, which might be due to the relatively lower activation energy for C–H bond scission facilitated by the catalytic properties of the material. Therefore, to further improve the yield of gaseous products, including hydrogen, Shen *et al*. [29] performed the microwave-assisted pyrolysis of high-density polyethylene (HDPE) using iron-based catalysts (Fe@SiC, Fe@AC and Fe@SiO_2_) in a multi-mode microwave oven (2.45 GHz) at 800 W. Overall, Fe@AC exhibited the highest permittivity, followed by Fe@SiC, whereas Fe@SiO_2_ showed the least value. As a result, Fe@AC showed the maximum affinity towards gases (H_2_ yield = 83.5%) and solid carbons, owing to its higher dielectric loss (tan δ = 2.44). The authors attributed the high hydrogen yield to the formation of hotspots on the Fe catalysts due to their high dielectric loss, which facilitated the cracking of higher hydrocarbons into lighter gases and carbon. Similarly, the morphology and the average diameter of the carbon nanotubes (CNTs) produced directly correlates with the dielectric properties of the iron-based catalyst. For instance, Fe@AC produced CNTs with an average diameter of 141 nm whereas Fe@SiC resulted in notably smaller diameter of CNTs (26 nm), suggesting the growth of CNTs under rapid heating over Fe@AC.

Yao *et al*. [30] studied the microwave-assisted decomposition of HDPE over Fe/FeAl_2_O_4_. Briefly, FeAl_2_O_4_ was loaded with different wt% of Fe, where an increase in Fe loading led to a decrease in the average crystal size of FeAl_2_O_4_. Also, it resulted in an increase in the imaginary part of the permeability (*μ*″) from 0.301 to 0.345 at 2.45 GHz. This indicates that a higher Fe content enhanced the catalyst’s ability to convert electromagnetic energy into thermal energy, as evidenced by the increase in reaction temperature from 140℃ to 330°C at 300 W. Similarly, an increase in microwave power subsequently reduced the reaction time from 34 to 6 min whereas no reaction occurred at lower microwave power (200 W, 180°C). The authors further describe the heating mechanism by noting that the magnetic field around the Fe/FeAl_2_O_4_ induces eddy current loss, with Fe serving as the primary microwave-absorbing point. This localized heating is then transmitted to FeAl_2_O_4_, resulting in an overall increase in the temperature. Overall, the results revealed that the 30 wt% Fe/FeAl_2_O_4_ catalyst effectively deconstructed the HDPE into H_2_ (47.03 mmol/g_HDPE_) with a concentration of approximately 85 vol%.

A slightly higher H_2_ yield (50.2 mmol/g_plastic_) was reported by Wang *et al*. [31] while performing the microwave-assisted pyrolysis of HDPE over a tandem catalyst (Fe/Ni-CeO_2_ @CNTs). In a nutshell, the use of CNTs significantly improved the heating rate (17.8°C s^−1^) because of its higher microwave absorption capabilities, whereas the use of bimetallic Fe/Ni nanoparticles triggered the C–H bond scission. Similarly, the presence of CeO_2_ in the catalyst notably enhanced the H_2_ production (91.5 vol%), by facilitating the oxidation of carbon deposition and the reforming of reaction intermediates, driven by the transformation of Ce⁴^+^ and Ce³^+^. Overall, the catalyst showed exceptional activity and suggested a novel approach to producing high-quality H_2_ and value-added CNTs from waste plastics under microwave heating.

The activation of various ferrite-based bimetallic nanoparticles was compared by Shoukat *et al*. [32] who studied the activity of different catalysts, including NiFe_2_O_4_, ZnFe_2_O_4_ and MgFe_2_O_4_ for the microwave pyrolysis of HDPE at 450°C and at 1 kW. All the catalysts exhibited exceptional activity, with the extraction of 78% H_2_ in just 1 min. Compared with others, MgFe_2_O_4_ showed the highest activity and H_2_ yield (90%) due to its superior magnetic characteristics and reactivity. Also, it resulted in better quality and quantity of CNTs whereas both NiFe_2_O_4_ and ZnFe_2_O_4_ produced a mixture of CNTs and amorphous carbon.

High H_2_ yields were reported by Jie *et al*. [33] who performed the catalytic deconstruction of waste plastics using FeAlO_*x*_ in a microwave reactor with a maximum power output of 2 kW. The one-step method instantly (30–90 s) generated a high H_2_ yield (55.6 mmol/g_plastic_), which was close to the theoretical hydrogen content (97%) being extracted from plastics. By comparison, conventional pyrolysis exhibited a lower hydrogen yield (4.3 mmol/g_plastic_), with the maximum affinity towards oils (66%). This clearly illustrates the challenge of precisely activating C–H bonds in plastics to generate hydrogen using conventional heating methods.

Microwave-driven catalysis is a promising method to combat the problem of waste plastics and in parallel produce clean hydrogen and carbon nanotubes. The scalability of this method requires further investigation, to optimize efficiency. Similarly, to make the process more sustainable for H_2_ production, future research needs to analyse the economic costing and environmental impact associated with the process.

### (d) Hydrogen from ammonia

Ammonia is produced in vast quantities worldwide, predominantly due to its role in the production of fertilizers but also for its use in the chemical industry, for cleaning, refrigeration and steel production, among other uses. Over 180 M tonnes are currently made and transported worldwide per annum [34,35]. It is mostly produced via the Haber–Bosch process, which is responsible for 1.4% of the world’s carbon dioxide emissions and 1% of the world’s energy uses [36,37]. This high-energy usage explains the efforts being made to increase the efficiency of this process as well as the move towards its electrification [38].

Ammonia has also been widely investigated as an energy storage material with the goal of moving towards an ammonia-mediated hydrogen economy [39–42]. This is largely due to its high gravimetric and volumetric hydrogen density. Ammonia’s usefulness as an energy vector is also emphasized by the transportation network and infrastructure for ammonia already being in place. The use of ammonia for energy has the additional flexibility that it can either be burnt directly or decomposed to use the resulting hydrogen as the energy source.

#### (i) Catalytic synthesis of ammonia using microwaves

Due to the high energy demand of the Haber–Bosch process, there is a lot of interest in developing methods to reduce the energy demands of the ammonia production. Microwave-enhanced catalysis has been shown to be an effective method of reducing the energy requirements of ammonia synthesis. Wang *et al*. have demonstrated that ruthenium-based catalysts under microwave heating significantly reduce the required reaction temperatures compared with conventional heating [9]. This work demonstrated better ammonia production rates at higher pressures and lower H_2_/N_2_ ratios. Also shown was a temperature dependence with a peak somewhere in the range of 280–400°C depending on the gas flow rate. Ruthenium catalysts have also been shown to be effective at atmospheric pressure and lower temperatures with the addition of a support to adjust the loss tangent of the catalyst for more effective heating [43]. This study also showed that improvement in the ammonia production rate can be achieved with the addition of promoter ions such as potassium, cerium and barium. A key factor in the use of ammonia as a clean energy source is the ability to run using renewable energy sources that can face interruptions affecting ammonia production. The ammonia production rate using this method was not affected by interruption of power with the production rate staying consistent in both the stable and intermittent power regimes. Wildfire *et al*. found that increasing metal loading of the catalyst led to quicker response times to microwave heating allowing even better ease of use of this type of system under intermittent power from renewable energy sources [44]. Work looking into the use of CNTs as the microwave susceptor has shown that the catalyst preparation can have dramatic effects on the catalytic performance [45]. It was demonstrated that mechanical mixing of Cs−Ru/CeO_2_ catalyst with CNT increased the ammonia production over threefold compared with synthesis using co-precipitation and hydrothermal methods. This is due to the enhanced heating from improved dielectric properties promoting faster N_2_ dissociation.

**Figure 3.**
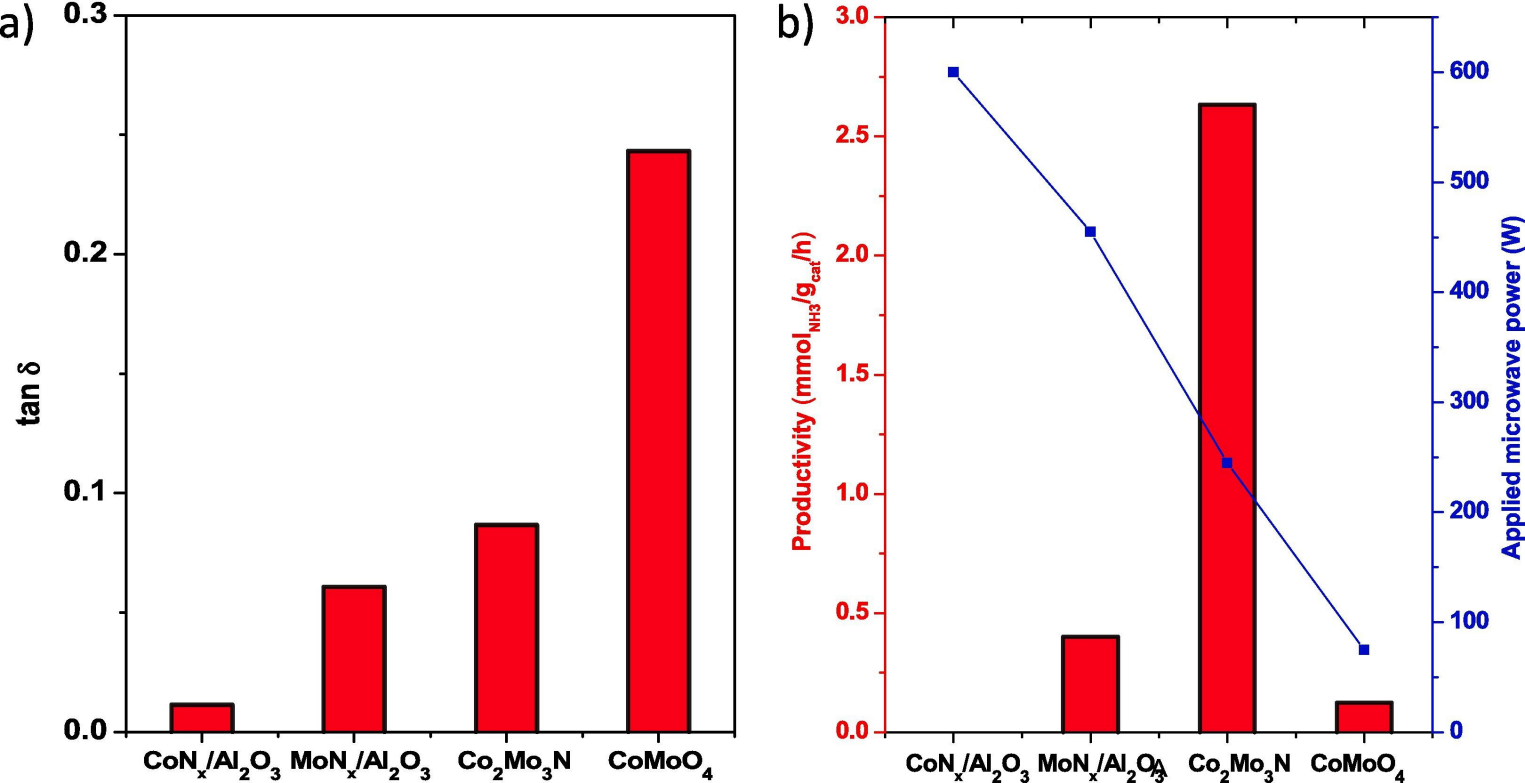
(*a*) Measurement of dielectric loss tangent of commercial-CoMoO_4_, Co_2_Mo_3_N, MoN_x_/Al_2_O_3_ and CoN_x_/Al_2_O_3_, (*b*) NH_3_ productivity (per mass) of commercial-CoMoO_4_, Co_2_Mo_3_N, MoN_x_/Al_2_O_3_ and CoN_x_/Al_2_O_3_ at 420°C, 7 bar pressure, under H_2_ and N_2_ partial pressure of 2:1, and 6900 h^−1^ GHSV in a continuous-flow reactor (coupled with a 2.45 GHz microwave reactor). Average power needed to reach 420°C: commercial-CoMoO_4_: 72 W, Co_2_Mo_3_N: 247 W, MoN_x_/Al_2_O_3_: 460 W and CoN_x_/Al_2_O_3_: did not heat even at 600 W [46].

Ruthenium is a very effective catalyst for ammonia production but is a rare earth metal leading to work using more earth-abundant metals. Dutta *et al*. have proposed the use of Co_2_Mo_3_N as a sustainable, cost-effective and more environmentally friendly alternative [46]. The effectiveness of this chosen catalyst is shown in figure 3, where the dielectric loss of the material allows it to be heated to 420°C using under 250 W as well as having very high catalytic performance compared with the other tested catalysts. The particle size of the catalyst used for ammonia decomposition has been found to be an important factor by Brown *et al*. [47].

As ammonia is produced in such vast quantities worldwide, an extensive scale-up of microwave catalytic techniques will be required for this technique to be useful as a clean energy solution. Melkote *et al*. have developed the largest reported lab-based microwave-driven ammonia reactor to date, showing a 10× scale-up in ammonia production from previous systems with a production rate of 56.6 g of ammonia per day [48]. As the authors note, although this increase in scale is significant, this work highlights the challenges of scaling up further using this type of microwave reactor. Increasing size can run into problems with microwave field distribution, microwave penetration depth, internal heat conduction and variations in temperature leading to impacted performance.

#### (ii) Plasma synthesis of ammonia using microwaves

**Figure 4.**
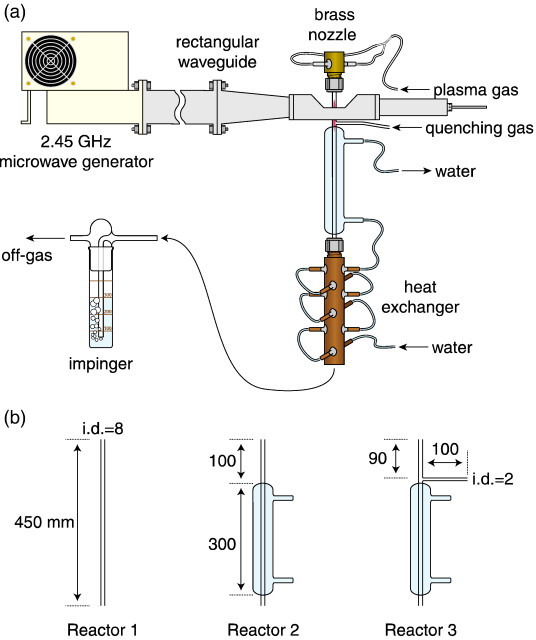
Schematic illustration of (a) experimental apparatus and (b) reactors [49].

The production of ammonia using a microwave plasma has also been suggested. Nakajima *et al*. investigated using a 1.3 kW microwave source at 2.45 GHz and a surfaguide field applicator acts as a resonant cavity to generate a microwave discharge [49]. This set-up is shown in figure 4 along with the three reactor designs investigated. This showed that water cooling of the reactor (reactor 2) showed very similar results to the uncooled reactor (reactor 1) due to the low thermal conductivity of the quartz tube. When a direct quenching gas was injected into the afterglow of the plasma, ammonia production rates were increased by up to a factor of 20 when compared with reactors 1 and 2 without quenching.

Plasma-enhanced catalytic chemical looping ammonia synthesis has been investigated by Brown *et al*. [50]. In this work, Fe, Mn and CoMo particles were pre-treated using microwave plasma before being used for standard thermo-catalytic ammonia synthesis and compared with traditional thermal systems. The nitrogen plasma pre-treatment was found to increase the reaction productivity and reduce required temperatures by pre-activating the nitrogen before it is deposited on the catalyst surface.

#### (iii) Catalytic decomposition of ammonia using microwaves

Another way in which microwaves are being used to produce clean energy related to ammonia is in their use to decompose ammonia into hydrogen and nitrogen so the hydrogen can be used as the fuel source. Using a catalyst with microwave heating at high temperatures, ammonia can be decomposed at bulk temperatures of 350–400°C at ambient pressure compared with 600°C required via conventional heating. This decreased reaction temperature is likely due to the formation of hotspots or ‘microplasmas’ within the catalyst bed during microwave heating. Some catalysts absorb microwave power less than others due to their dielectric loss. As the reaction temperature is created by microwave heating of the catalyst, for low loss catalysts the reaction temperature may not be reached with a reasonable amount of microwave power. In these cases, a microwave susceptor may be added to increase the catalyst temperature.

Seyfeli *et al*. investigated the use of both activated and mesoporous carbon-supported cobalt catalysts showing a slight performance increase in the mesoporous carbon samples at lower temperatures [51]. Also investigated is the performance of alumina versus activated carbon supported nickel-based catalysts with metal loading of 10 wt% [52]. In this work, the activated carbon-supported catalysts performed better under conventional heating whereas they were outperformed by the alumina catalyst during microwave heating. The catalyst was mixed as a 1:1 ratio with mesoporous carbon to reach 400°C required for the reaction.

The metal loading of catalysts is also an important factor to consider which was investigated by Dilek *et al*. with iron incorporated mesoporous carbon catalysts adjusting the metal loading from 5 to 15 wt% [53]. This showed that ammonia decomposition occurred at significantly lower temperatures with microwave heating versus conventional heating for all metal-loaded catalysts. The highest ammonia conversion among those tested in this work was found to be 7.7 wt%, likely due to the lower surface area and larger metallic crystals formed in this sample versus higher metal loadings. The addition of small amounts of rare-earth and alkaline-earth metals has also been studied. Catalysts based off these metals have been shown to be very effective but very expensive. Using small amounts of these metals allows high efficiency but keeps costs low. Yildiz *et al*. demonstrated that for all metal-promoted molybdenum catalysts tested with 4 wt% promoter added, microwave heating again outperformed conventional heating by requiring lower temperatures. At 350°C and above, all of the catalysts performed similarly, but below this temperature the barium and cerium showed better results than the calcium- and lanthanum-promoted catalysts [54].

**Figure 5.**
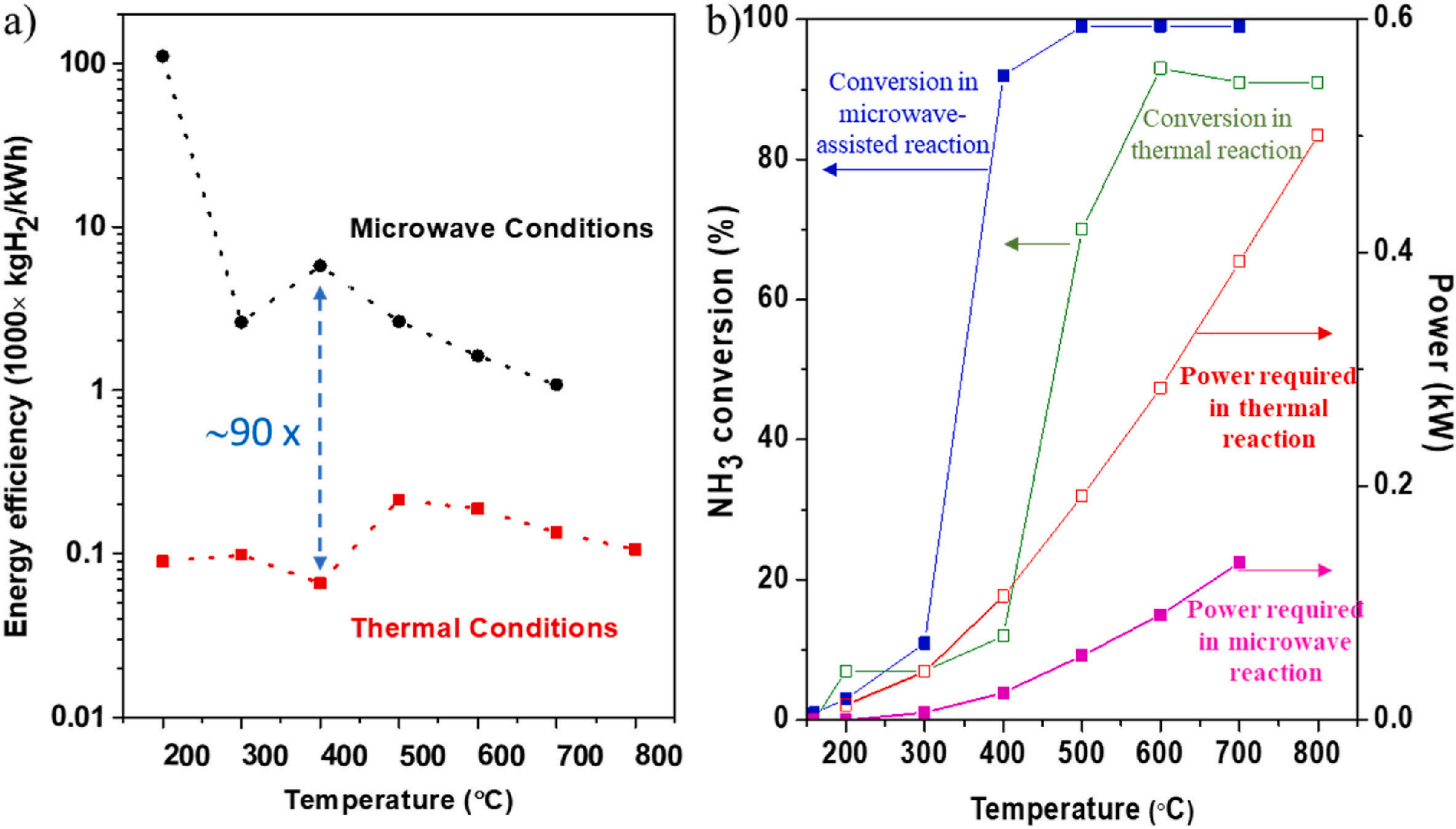
(a) Energy efficiency for NH_3_ decomposition reaction on Co_2_Mo_3_N under microwave and thermal reactions, and (b) energy (power) requirement to reach [55].

As shown in figure 5, microwave heating of a catalyst, in this case the metal nitride Co_2_Mo_3_N, to decompose ammonia can cause a higher conversion rate at lower temperatures as well as requiring considerably lower power. This leads to the dramatic increase of energy efficiency shown by Dutta *et al*. of 90 times improvement in efficiency for the microwave system compared with thermal conditions at 400°C [55].

#### (iv) Plasma decomposition of ammonia using microwaves

**Figure 6.**
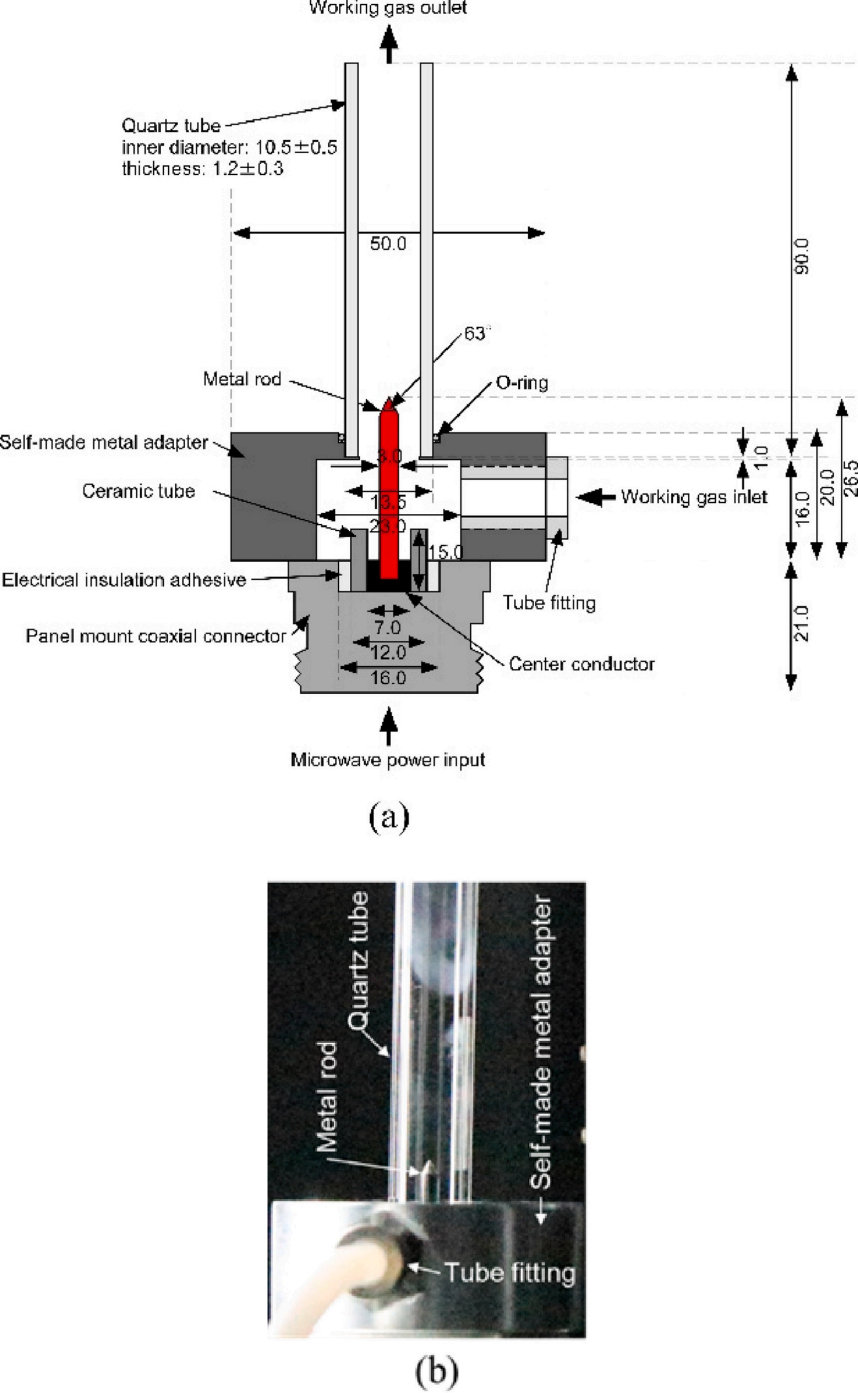
Structure diagram and photograph of rod-electrode-type microwave plasma system. (a) Structure diagram. (b) Photograph [60].

As well as using microwaves in reactors with a catalyst, microwaves have also been used to decompose ammonia using microwave-initiated plasmas. Dielectric barrier discharge plasma generators are a highly active area of research in this field [56–59], however, alternative microwave plasma generators have also been suggested in the literature. For example, Sekiguchi has developed a rod-electrode-type plasma source for the decomposition of ammonia [60]. The structural diagram for this applicator is shown in figure 6. This work posits that the high cost of catalytic decomposition with or without plasma can be hampered by high catalyst cost and/or high heating time. Direct dielectric barrier discharge (DBD) decomposition can be used without a catalyst but has low yields. The aim of this work was to develop a method for on-site rapid decomposition of ammonia with a high decomposition ratio. Using this rod-electrode-type plasma source developed in this work, and a slow flow rate of 0.2 l min^−1^, a hydrogen yield of approximately 90% was achieved. Zhang *et al*. proposed the use of a microwave argon plasma jet for ammonia cracking [61]. This initial work on this method of cracking ammonia shows promise as the very high gas temperatures of approximately 4500 K was a very effective method of decomposing ammonia, but more work was required to optimize hydrogen production rate and efficiency.

#### (v) Microwave measurements of ammonia

Microwaves have also been shown to be an effective way of measuring ammonia and characterizing ammonia storage materials. As ammonia is a very polar molecule with a high dielectric loss, the microwave cavity perturbation (MCP) technique has been used to measure the complex permittivity of materials as ammonia is absorbed and desorbed from the sample. The MCP technique allows a non-contact, non-invasive, non-destructive and *in situ* measurement of dielectric properties of the sample. The method allows for sampling rates of up to 1 kHz to be taken without loss of sensitivity [62]. This tracking of permittivity has been shown to be an accurate measure of the quantity of ammonia absorbed by the sample.

MCP has also been combined with structural material characterization via neutron diffraction as a method of assessing materials for their suitability for ammonia storage in the context of energy storage. The combination of these two spectroscopic techniques has been shown to give more information than either technique individually, where physisorbed and metal-coordinated ammonia can be differentiated [63]. This work has shown that group two halides such as MgI_2_ and CaBr_2_ [63] as well as metal-organic frameworks such as UiO-67 [64] show promise as future ammonia storage materials.

#### (vi) On the adoption of microwave technologies in industry

The adoption of microwave technology for hydrogen production faces challenges such as installation costs and variabilities in central government energy policies across different countries [65,66]. Consequently, the global industrial future of microwave technology is currently uncertain, as it depends upon country-specific priorities and access to energy resources such as fossil fuels. In general, for countries having excess fossil fuel resources, energy costs do not play a dominant role in investment decisions. This review underscores the application of microwave technology in clean energy production, where the primary goal is to minimize the dependency on fossil fuels. Here, energy costs become a critical factor, making industries more open to solutions offered by innovations including microwave technology. Nevertheless, a thorough cost benefit analysis is still at the heart of important decisions regarding the large-scale adoption of microwave technology. Despite high initial costs, microwave technologies offer a number of well-established advantages that support their scalability in industrial applications:

— *Energy efficient*. Microwave heating minimizes energy wastage by focusing heat directly on the reactants through mechanisms such as ion conduction and dipole polarization. This allows reduced reaction temperature and time that make the process potentially more energy-efficient overall [67].
— *Selective heating*. As discussed in previous sections, microwave heating is characterized by selective generation of heat in the desired regions of the material. For instance, electromagnetic waves selectively interact with catalysts during processes like hydrogen production from waste plastics. This interaction rapidly heats the catalyst particles, promoting catalytic cracking of carbon–hydrogen bonds and enhancing hydrogen yield [68].
— *Reduced processing times*. Higher reaction rates greatly reduced the processing time compared with conventional processes. This eventually leads to higher throughput and increased productivity [69].
— *Lower bulk temperatures*. Microwave heating relies on the material’s complex permittivity or permeability. A varying complex permittivity inside the feedstock itself can lead to localized heating. This localized heating intensifies the process while maintaining a lower bulk temperature. Thus, enabling easier handling of materials often leads to decreased plant footprint as auxiliary equipment that typically handles hot gases is not necessary [15].
— *High purity*. The rapid heating mechanism associated with microwaves minimizes undesirable side reactions, ensuring high purity of the material [70].
— *Lower maintenance cost*. Microwave heats the target material and not the surroundings, and therefore maintenance cost remains relatively low compared with conventional systems [71].
— *Catalyst optimization*. Using cost-effective, non-precious metal catalysts that retain high performance under microwave heating could be key to large-scale adoption. As discussed in previous sections, a sustainable and cost-effective Co_2_Mo_3_N catalyst for ammonia production as reported by Dutta *et al*. [46] has been found more effective to use with microwaves due to its optimum dielectric properties.

Despite these advantages, installation and running costs alone are not sufficient to justify the adoption of microwave technology, particularly when it is compared with well-established processes. Ultimately, factors such as product quality that cannot be achieved by existing means, process intensification and total electrification (no requirements for fossil fuel infrastructure) and flexibility of installation (installation in remote areas or countries that have electricity supply but limited infrastructure) have a more important role to play in future applications.

## 3. Microwaves in new battery technologies

Traditional synthetic routes for battery cathode and anode materials include solid-state ceramic, sol-gel and hydrothermal and solvothermal methods [72–77]. By comparison, microwave and microwave-assisted synthetic routes have shown themselves repeatedly to be faster, more efficient approaches [78–80].

### (a) Cathode materials

Compared with anode materials, cathode materials tend to be the limiting factor for the overall specific capacity of a cell. There is, therefore, a comparatively much stronger drive in battery research to identify suitable cathode materials for lithium-ion batteries (LIBs). LiCoO_2_ was one of the earliest cathode materials used in LIBs and remains among the highest performing in commercial cells. This is largely due to its high theoretical capacity (274 mA h g^−1^) and excellent mechanical stability [81]. Despite this, however, there is a need to move away from this material due to ethical, environmental and economic concerns surrounding the sourcing of cobalt [82,83]. As such, a large focus of cathode material research activities is in identifying potential materials that either reduce or eliminate the amount of/need for cobalt [81,82].

#### (i) Transition metal phosphate cathodes

Cathode materials based on transition metal phosphates, such as lithium-iron phosphate (LiFePO_4_), are an economic and non-toxic alternative to cobalt-rich materials [84,85]. They have been recognized as having good thermal and mechanical stability, high specific charge capacity and good cycling performance [86]. Materials within this family will have the general form LiMPO_4_, where M can be Ni, Mn, Co or some combination of all three.

Current efforts in the literature largely focus on the development of nanostructured materials with a view to improving overall performance. Having a smaller average particle size can reduce average diffusion path length while the larger surface area improves the electrode to electrolyte contact [87,88]. Thermal/mechanical stability is vital to achieving good, safe cycling performance [85]. Achieving higher specific capacities and increased redox potentials helps to drive down the overall mass (and cost) [83], a particularly important area for the rapidly developing electric-automotive industry.

Reported microwave synthetic routes for olivine transition metal phosphate cathodes are typically a co-precipitation reaction of metal salts held either in suspension (hydro-/ solvo-/iono-thermal) or through a solid-state process [89,90]. This is notably a single-step reaction, and frequently the entire synthesis can be carried out inside the microwave reactor with the final product being collected and washed at the end.

Process conditions can play an important role in determining final product quality. Ashton *et al*. investigated the diffusion behaviour of Li^+^ in nanostructured olivine LiFePO_4_ using muon spin relaxation spectroscopy (µSR) [91]. In this study, nanoparticulate of LiFePO_4_ was synthesized by a microwave solvothermal process using two different solvents: ethylene glycol and 1-ethyl-3-methyl imidazolium (EMI-TFMS). Though it was previously reported that the choice of solvent influences the phase of nanocrystalline LiFePO_4_ formed [92], this was the first time muon spin relaxation had been used to examine the effect of these on Li^+^ diffusion. µSR showed that while the nanocrystalline *Pnma* LiFePO_4_ phase exhibited similar diffusion coefficients to bulk LiFePO_4_, the presence of the higher pressure *Cmcm* phase LiFePO_4_ impeded Li^+^ mobility, leading to reduced diffusion. Importantly, the proportion of *Cmcm* phase LiFePO_4_ formed was found to be due to a combination of factors including reactants, and process temperature in addition to the choice of solvent. The frequency-dependent dielectric properties of these solvents used was shown to be highly illuminating in determining the causes of these reaction pathways.

**Figure 7.**
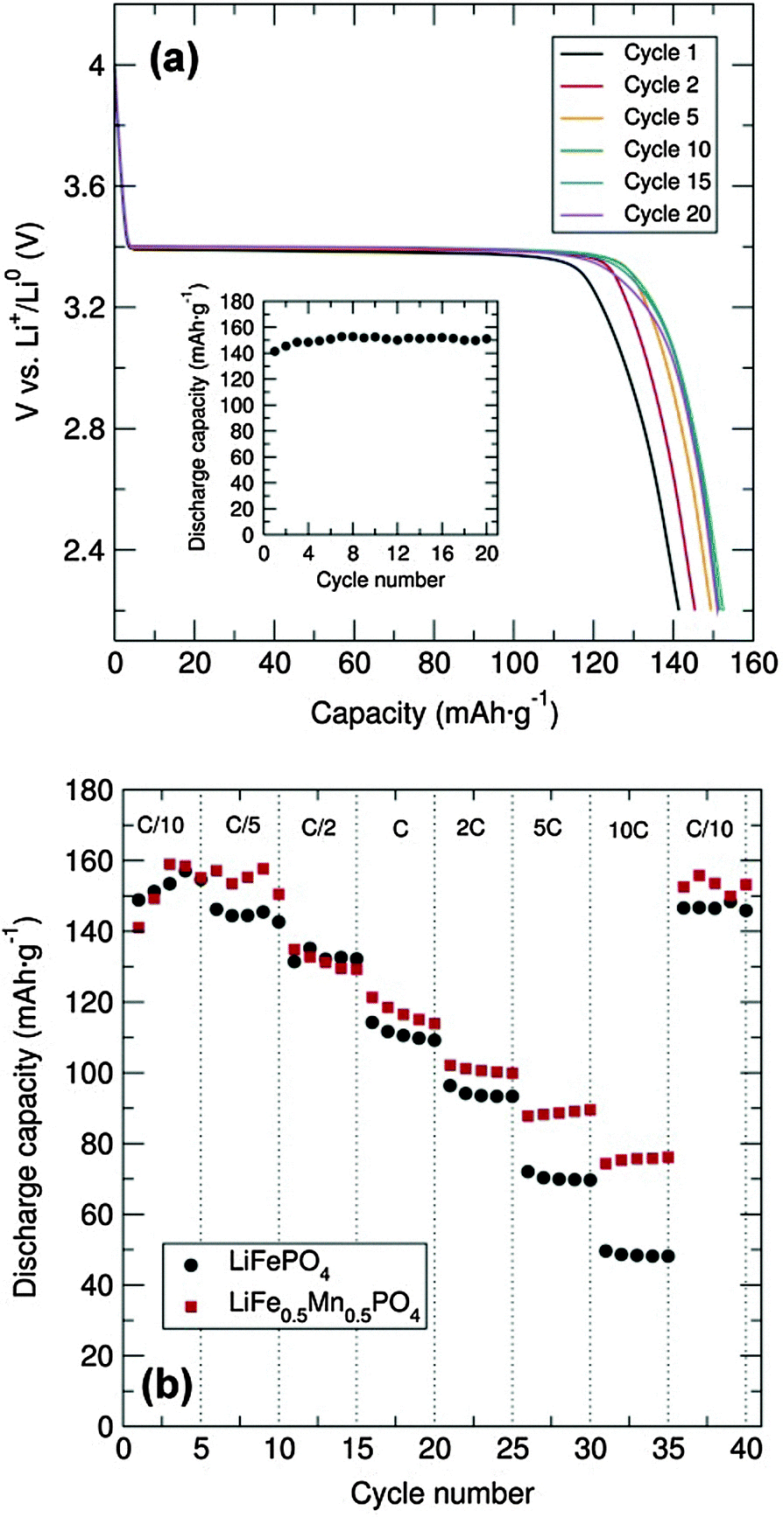
(a) Cycling performance and capacity fading (inset) of C/LiFePO_4_ prepared from [Fe(O^t^Bu)_2_(THF)]_2_ alkoxide precursor (mixed with C black and PTFE in 60 : 30 : 10 weight%) between 2.2 and 4.0 V at C/20 rate. (b) Rate performance of C/LiFePO_4_ and C/LiFe_0.5_Mn_0.5_PO_4_ (mixed with C black and PTFE in 60 : 30 : 10) at different charge–discharge C rates [94].

One method to improve the properties of these materials reported is the partial substitution of Fe by Mn. This leads to increased redox potential due to the higher Mn^2+/3+^ potentials as compared with the Fe^2+/3+^ [93]. Laveda *et al*. reported the fast microwave synthesis of mixed metal LiFe_1-*x*_Mn_*x*_PO_4_ [94]. Their approach made use of single source alkoxides, the benefit of which is that as a starting material all or most of the desired metals are located within a single compound with the same stoichiometry. They successfully obtained powdered, olivine structures of LiMPO_4_ (M being shorthand for Fe and Mn) after just 10 min of microwave treatment. The electrochemical performance of their C/LiFe_0.5_Mn_0.5_PO_4_ materials showed improved charge/discharge capacities of 75 mA h g^−1^ at a rate of 10C compared with the approximately 55 mA h g^−1^ at only 8C reported by Tripathi *et al*. for materials prepared via electrospun synthesis [80]. Most notable in their results was the near total recovery of capacity when cycling back at C/10 rates as shown in figure 7.

**Figure 8.**
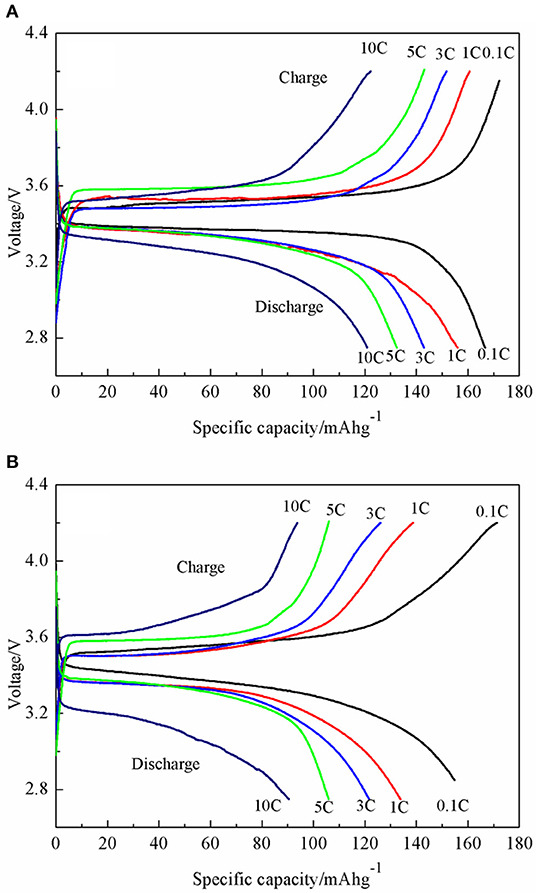
Charge/discharge profiles for (A) LiFePO_4_/graphene and (B) LiFePO_4_/C [99].

**Figure 9.**
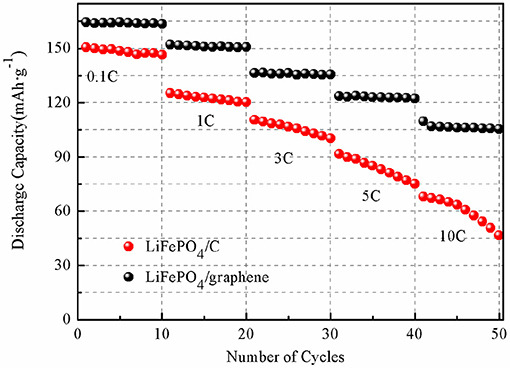
Cycling performance of LiFePO_4_/graphene (black) and LiFePO_4_/C (red) [99].

While the use of graphene to construct composite materials is common practice in the synthesis of anodes [95] including by microwave methods [96–98], there has been some work demonstrating their use for cathode materials. The synthetic route is typically the reduction of graphene oxide (GO) to form reduced graphene oxide (rGO). The abundance of functional oxygen groups on the surface of GO makes it possible to attach nanoparticles of other functional materials (commonly transition metal oxides) [95]. Liu *et al*. reported the synthesis of LiFePO_4_/graphene micro and nanoscale composites for use as cathode material using a one-step microwave heating method to perform the reduction of GO [99]. In their process, they combined a solution of FeSO_4_·7H_2_O and H_3_PO_4_ in deionized water and ethylene glycol, with a LiOH·H_2_O aqueous solution and GO suspensions maintaining a molar ratio for Li:Fe:P of 3:1:1. After stirring, this mixture was evaporated and pressed to form pellets. These pellets were then heated in a 1.5 kW microwave oven to perform the reduction and produce the LiFePO_4_/graphene composite. XRD results confirmed the complete reduction of GO and showed good phase purity of the LiFePO4 *Pnma* phase group. Electrochemical results were very promising, with an initial specific capacity of 120.9 mA h g^−1^ measured for 10C. This figure did fall off initially during subsequent charge–discharge cycling but remained otherwise consistent over the full 10 cycles as shown in figures 8 and 9.

#### (ii) Transition metal oxide cathodes

Transition metal-layered oxide cathodes are typically of the form LiMO_2_ where M can be Ni, Mn, Co or some combination of all three such as, for example, LiNi_1/3_Mn_1/3_Co_1/3_O_2_ (NMC111). Ni-rich variants (such as NMC811, NMC622) are particularly attractive as their high energy density makes them well suited for the electric vehicle (EV) market [100,101].

**Figure 10.**
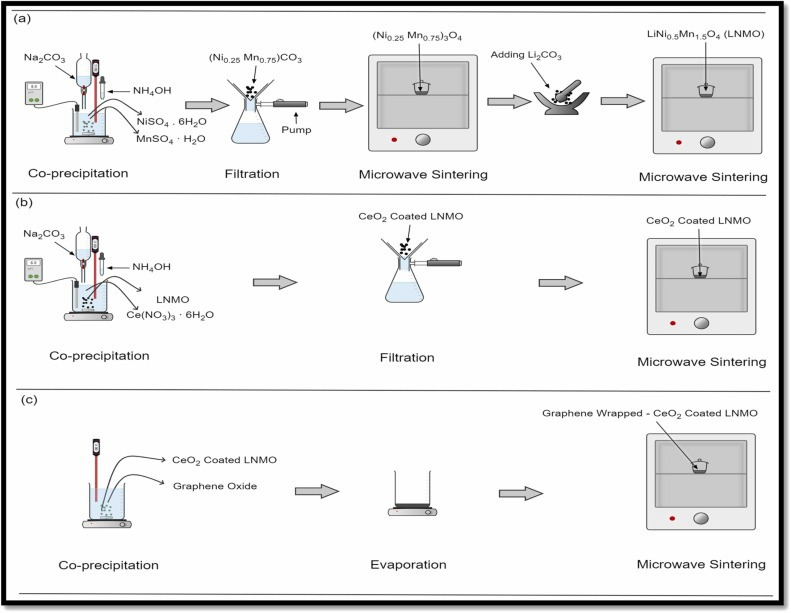
Microwave-assisted synthesis of LiNi_0.5_Mn_1.5_O_4_. A conventional co-precipitation is performed, with the final calcination step undertaken in a microwave furnace [102].

The synthetic route for these materials is generally a co-precipitation of stoichiometric amounts of transition metal salts through the addition of a base to form a hydroxide precursor. This precursor is then calcined with a suitable source of lithium to form the final layered oxide [81]. Microwave methods vary and have been used for different steps of the reaction. Frequently, the conventional approach is used to produce the hydroxide salt, with the final calcination performed by microwave furnace [102–104] as illustrated in figure 10. Alternatively, a sol-gel approach can be employed [105,106]. In these approaches, microwaves have been used alternately both to form the precursor sol-gel [106] itself and to perform the final calcination step [105].

The faster synthetic routes offered by microwave methods can allow significant influence over the final morphology [91,94,107]. In the synthesis of Ni-rich NMC variants, the precise control of temperature and treatment time is vital during the stabilization of Ni3+ during calcination [105].

Feng *et al*. directly compared the performance of LiNi_0.5_Mn_1.5_O_4_ cathode materials produced by microwave and conventional heating [106]. They prepared their samples following a sol-gel approach to form a porous xerogel through calcination using both microwave and conventional heating. Both sample types were then sintered by conventional heating to form the final cathode material. They found that the rapid heating in the microwave process was more effective at removing crystalline water in the final product, yielding a purer and more stable material leading to better electrochemical performance. The specific capacity of the microwave-treated sample was found to be 122 mA h g^−1^ at 10C charge rate, far surpassing that of the conventionally heated sample, which was only 69 mA h g^−1^. Cycling performance was also seen to be dramatically improved in the microwave sample.

Another direct comparison between microwave and conventional synthesized LiNi_0.5_Mn_1.5_O_4_ was presented earlier by Gao *et al*. [104]. Here, they followed the conventional co-precipitation of the hydroxide salt, with the final calcination/sintering step being performed by microwave and conventional heating for comparison. Similar to the results of Feng *et al*. [106] they saw reduced impurities in the microwave-prepared samples, leading to enhanced electrochemical performance. Their microwave-prepared samples also outperformed the conventionally prepared ones, with 108.7 mA h g^−1^ versus approximately 85 mA h g^−1^ at 10C charge rates.

Similar to work done previously for LFP [91], Johnston *et al*. [105] reported localized diffusion characteristics of Li^+^ in nickel-rich layered oxide cathodes using µSR. Using this technique, they examined the effect of doping on Li^+^ diffusion behaviour in NMC cathode materials produced by microwave sol-gel synthesis. They found that even small amounts of mechanically stabilizing dopants, such as Al or Mg, can hinder the ionic diffusion of Li^+^.

### (b) Anode materials

Anode materials for LIBs are frequently carbon-based, largely because of their layered structure and good electrochemical compatibility with lithium electrolytes that enable the reversible intercalation of Li ions. Graphite was one of the first materials used for LIBs [85,108], making it possible to use intercalating cathode materials for the first time without the need for potentially dangerous lithium metal as the anode. Thanks to its theoretical capacity of 350 mA h g^−1^ (370 mA h g^−1^ once cycled sufficiently to form LiC_6_) [85,109], graphite still remains widely used as anode material. Despite this, however, there is a variety of new materials emerging, some carbon-based, and some not. This is especially true in the field of sodium ion batteries due to the lack of suitably sized intercalating zones in graphite for the (comparatively) much larger Na^+^ ion.

#### (i) Reduced graphene oxide anodes

The discovery of graphene in 2004 was hugely influential in the development of new anode materials, most notably those based on rGOs. As was discussed briefly in the previous section, the abundance of functional oxygen groups on the surface of GO makes it possible to attach nanoparticles of other functional materials (commonly transition metal oxides) [95,98]. This makes it very attractive as a base for synthesizing a variety of functionalized anode materials.

The microwave synthetic route for rGO-based anode materials typically starts with the synthesis of GO itself. This is conventionally performed by a modified Hummer’s method using naturally sourced graphite [98,110]. Once GO has been synthesized it is usually suspended in deionized water, at this point if a composite is being produced a solution of these precursors would then be introduced in the requisite stoichiometric quantities [95]. Finally, the hydrothermal reduction is performed inside the microwave reactor. The resultant precipitate is collected, washed, dried and then typically calcinated under an inert atmosphere (i.e. N_2_, Ar).

Since rGO networks are excellent hosts for metal nanoparticles, they are frequently used to provide mechanical and electrical stability to otherwise promising metal oxides in an effort to better leverage their theoretical capacity. Kang *et al*. successfully synthesized CuCo_2_O_4_ nanocubes with an rGO outer layer for use as anode material. They reported greater discharge capacities of 570 mA h g^−1^ after 350 cycles, claiming to outperform results for conventionally synthesized, non-composite CuCo2O4 reported by Sharma *et al*. [111]. Gangaraju *et al*. used a similar process of microwave reduction of GO to produce graphene-carbon nanotube-Mn3O4 ‘nanoalloys’ for use as anodes in LIBs, achieving 1337 mA h g^−1^ after 300 cycles.

## 4. Outlook and conclusions

This review has highlighted important recent advances and promising potential for future developments of microwave applications in hydrogen energy and new battery technologies. Key findings from the studies reviewed here indicate that microwave technology can enhance the efficiency, reaction rates and overall effectiveness of hydrogen-producing processes and battery material synthesis protocols, offering unique advantages over conventional methods.

The use of microwaves for catalytic water splitting shows substantial promise, with localized heating enabling lower bulk temperatures and enhanced reaction rates. The development of CGO catalysts has demonstrated significant potential in producing hydrogen efficiently at temperatures below 250°C. This method’s energy efficiency, coupled with the economic feasibility of microwave-assisted hydrogen production, positions it as a competitive alternative to traditional electrolysis methods. Microwave-assisted catalytic decomposition of hydrocarbons, such as methane and paraffin wax, has been shown to achieve high conversion rates and hydrogen yields. Innovations in catalyst design, including the use of Fe–Ni alloys and ruthenium nanoparticles, have significantly improved the efficiency and selectivity of these processes. Microwaves have proven effective in both the synthesis and decomposition of ammonia, offering a pathway to reduce the energy demands of the Haber–Bosch process and improve hydrogen production efficiency. Ruthenium-based catalysts, as well as more sustainable alternatives like Co_2_Mo_3_N, have shown enhanced performance under microwave heating.

Microwave-assisted synthesis of cathode materials for batteries has been particularly effective for transition metal phosphate and oxide cathodes, such as LiFePO_4_ and LiNi_0.5_Mn_1.5_O_4_. The rapid and uniform heating provided by microwaves leads to high-purity materials with superior electrochemical performance. The development of nanostructured and composite materials, including those involving graphene, further enhances battery capacities and cycling stability. For anode materials, the use of rGO and its composites in anodes has been a significant focus, leveraging the mechanical and electrical stability of rGO to support high-capacity metal oxides. Microwave synthesis has enabled the production of high-performance anode materials, such as CuCo_2_O_4_ and Mn_3_O_4_ composites, demonstrating substantial improvements in capacity and cycle life.

The integration of microwave technology in hydrogen generation and battery materials synthesis represents a significant leap forward in clean energy solutions. The ability to achieve rapid, selective heating and enhanced reaction rates opens new avenues for efficient and scalable energy production. Our understanding of the complex mechanisms of microwave interaction has been advancing rapidly in recent years, and ongoing research and innovation continue to bridge the gap between theory and practice, driving further advancements in the field.

## Data Availability

No new data was created during this study.
